# Comparative study on proximate, functional, mineral, and antinutrient composition of fermented, defatted, and protein isolate of *Parkia biglobosa* seed

**DOI:** 10.1002/fsn3.373

**Published:** 2016-04-29

**Authors:** Bolajoko I. Ogunyinka, Babatunji E. Oyinloye, Foluso O. Osunsanmi, Abidemi P. Kappo, Andrew R. Opoku

**Affiliations:** ^1^ Biotechnology and Structural Biochemistry (BSB) GroupDepartment of Biochemistry and MicrobiologyUniversity of ZululandKwaDlangezwa3886South Africa; ^2^ Department of BiochemistryCollege of SciencesAfe Babalola UniversityPMB 5454Ado‐Ekiti360001Nigeria

**Keywords:** Antinutrient, functional properties, *Parkia biglobosa*, protein isolate, proximate analysis

## Abstract

The use of plant‐derived foods in the prevention, treatment, and management of metabolic diseases especially diabetes has gained prominence; this has been associated with their physicochemical properties. This study was conducted to compare the proximate, functional, mineral, and antinutrient composition of the fermented seeds, the defatted seeds, and the protein isolate from *Parkia biglobosa* seeds. The results showed that the fermented, defatted, and protein isolate varied in composition within the parameters studied. The proximate analysis revealed that the protein isolate had the highest ash (6.0%) and protein (59.4%) as well as the lowest fat (5.7%) and moisture (5.1%) content when compared to the fermented and defatted samples. In like manner, the functional properties of the protein isolate were relatively better than those of the fermented and defatted samples, with oil absorption capacity of 4.2% and emulsion capacity of 82%. The magnesium and zinc content of the protein isolate were significantly higher when compared with the fermented and defatted samples, while a negligible amount of antinutrient was present in all the samples, with the protein isolate having the lowest quantity. The overall data suggest that the protein isolate had better proximate, mineral, functional, and antinutrient properties when compared to the fermented and defatted samples. Therefore, the synergistic effect of all these components present in the protein isolate from *P. biglobosa* seed in association with its low carbohydrate and high protein/ash contents could play a vital role in the management of diabetes and its associated complications.

## Introduction

*Parkia biglobosa* is one of the indigenous medicinal plants commonly found in Nigeria and many other West African countries. The fermented seeds of *P. biglobosa* are believed to possess nutritional and medicinal values; hence, they are used extensively as household spice in the preparation of various delicious foods (Ajaiyeoba 2002; Ogunyinka et al. 2015). *P. biglobosa* belongs to the family *Leguminosae* and subfamily *Mimosoideae*. It is known as African locust beans (English); “dawadawa” in Ghana; “soumbala” in Burkina Faso; and “iru” in Benin and Nigeria (Pelig‐Ba 2009).

In recent times, several pharmacological and nutritional benefits of *P. biglobosa* have been reported (Badu et al. 2012; Olabinri et al. 2013). Consequently, considerable interest has been directed toward exploring *P. biglobosa* in the prevention, treatment, and management of a number of metabolic diseases among which is diabetes mellitus. This pharmacological and nutritional benefit of *P. biglobosa* has been associated with its physicochemical properties, which is perceived to act independently or in synergy; to play a pivotal role in the maintenance of normoglycemia by activating diverse protective mechanisms (Fred‐Jaiyesimi and Abo 2009; Ogunyinka et al. 2015).

Diabetes is a multifactorial disease connected with numerous complications, and consequently requires multiple therapeutic approaches (Modak et al. 2007). *P. biglobosa* has been identified as one of the candidates with promising therapeutic potential (Odetola et al. 2006). As part of exploration into the protective and antidiabetic mechanisms of *P. biglobosa*, this study was carried out to compare the proximate, functional, minerals, and antinutrient composition of the fermented, defatted, and protein isolate of *P. biglobosa* seeds.

## Materials and Methods

### Plant material, authentication, and extract preparation

After obtaining import permit (P0060156) from the Department of Agriculture, Forestry and Fisheries (DAFF, Republic of South Africa); raw and fermented *P. biglobosa* seeds used in this study were purchased from a local market in Ijebu‐Ode, Ogun state, Nigeria. The raw seeds were identified and authenticated by the Chief botanist of the Department of Botany, University of Zululand and a voucher specimen (B07) was deposited in the University Herbarium. Prior to separation of protein isolate, the fermented seeds were oven‐dried at 50°C; thereafter, the dried seeds were grounded into uniform powder using an electric blender. One kilogram of uniform powdered seeds was defatted with hexane to obtain the defatted extract.

The defatted extract was air‐dried and thereafter extracted (1:10 w/v) with acidic butanol to remove possible antinutrients. Protein isolate was obtained from the defatted extract using the method described by Nkosi et al. (2005). The dry defatted extract was resuspended in distilled water at pH 10. The resultant suspension was filtered to remove debris and the filtrate adjusted to pH 5, followed by centrifugation at 7650 × g for 15 min at 4°C. The supernatant from this centrifugation step was discarded while the pellet containing the protein isolate was retained and freeze‐dried to yield a brown extract. The lyophilized extract was kept dry until needed.

### Proximate analysis

Proximate analysis was carried out on the fermented *P. biglobosa* seeds, the defatted *P*. *biglobosa* seeds (DPB) and protein isolate from *P. biglobosa* seeds (PBPI) to ascertain the differential composition of the various nutritional components and the amount in which they are composed. Nutritional parameters such as ash content, moisture content, mineral composition, crude fat, carbohydrate, crude fiber, and total protein were determined. The official method of AOAC (2005) was used to determine the moisture contents, while Tecator Digestion System and Kjeltec Auto 1030 Analyzer (Tecator AB, Sweden) was used to determine the protein contents. The Soxtec System HT method (Tecator Soxtec System HT 1043 Extraction Unit, Tecator AB, Sweden) was used to determine the fat contents. The AOAC (2005) official method was used to determine the ash contents. The crude fiber contents were determined in accordance with AOAC (2005). The value of 5.95 was used as protein conversion factor during calculation. Protein yields were thus calculated as follows:

$$\text{Yield}(\%)=\frac{\text{weight}(g)\text{of PBPI}\times \text{protein content}(\%)\text{of PBPI}\times 100}{10g(\text{weight of DPB})\times \text{Protein content}(\%)\text{of DPB}}$$

The carbohydrate content was determined by difference, after addition of all the percentages of moisture, fat, crude protein, ash, and crude fiber and was subtracted from 100%. This gave the amount of nitrogen‐free extract otherwise known as carbohydrate content.

Carbohydrate(%)=100−[protein(%)+Moisture(%)+Ash(%)+Fiber(%)+Fat(%)].

The calorie value of the samples in Kilojoules per 100 g (kJ/100 g) was determined using the “Atwater factors” by multiplying the value of crude protein, lipid, and carbohydrate by 37, 17, and 17, respectively (Eknayake et al. 1999).

### Mineral analysis

The official method of AOAC (2005) was adopted for the mineral analysis of the samples: the samples were previously ashed in a furnace for 5 h at 600°C, and then refluxed with 20% hydrochloric acid. The mixture was filtered into a 100 mL standard flask; the filtrate was then made up to the mark with deionized water. Sodium (Na) and potassium (K) levels of the samples were ascertained using a flame emission photometer with NaCl and KCl as standards. All other metals were determined by atomic absorption spectrometry (AAS).

### Antinutritional composition

The antinutrient contents of fermented, defatted, and protein isolate of *P. biglobosa* seeds were determined. Phytate was determined by the method described by Ola and Oboh (2000), while oxalate level was ascertained using the method described by Krishna and Ranahan (1980).

## Functional Properties

Functional properties of fermented, defatted, and protein isolate of *P. biglobosa* were evaluated as follows:

### Bulk density

A previously marked 10 mL measuring cylinder was filled with 2 g of the samples. The measuring cylinder base was gently tapped on the laboratory table severally to a constant volume; bulk density (g/mL) was calculated by the method of Akpapunam and Markakis (1981), using the formula:

$$\text{Bulk density}=\frac{\text{Weight of samples}}{\text{volume of sample after tapping (g/mL)}}.$$

### Swelling index

Swelling index was evaluated using the method described by Akinyele et al. (1986). One gram (1 g) of the samples was weighed and dispersed into a test tube, leveled, and the height was noted. Distilled water (10 mL) was added and allowed to stand for 1 h. The height was then recorded and the swelling index was calculated using the formula:

$$\text{Swelling index (S)}=\frac{\text{Initial height (H1)}}{\text{Final height (H2)}}$$

### Water absorption capacity

Water absorption capacity was determined by measuring 1 g of the samples and then mixed with 10 mL of distilled water in a weighed 20 mL centrifuge tube. The suspension was vigorously mixed with a vortex mixer for 2 min, after which it was rested for 30 min. Thereafter, the suspension was centrifuged at 4973 × g for 25 min. The supernatant was discarded and the tube was air‐dried. Bound water was calculated from the increase in the weight of the samples (Sathe and Salunkhe 1981). The water absorption capacity (WAC) was calculated using the formula:

$$\text{WAC}\;=\text{Initial volume}\;\left(V_{1}\right)-\frac{\text{Final volume}\;(V_{2})\times 1.0}{100}$$

### Oil absorption capacity

Five hundred milligram (0.5 g) of the samples was added to 3.0 mL of corn oil in a 15 mL conical graduated centrifuge tube. The emulsion were mixed with a vortex mixer for 2 min, incubated at room temperature for 30 min, and later centrifuged at 4896 × g for 25 min. The supernatant was decanted and the volume of separated oil was noted (Sathe and Salunkhe 1981). Oil absorption capacity (OAC) was evaluated by difference using the formula:

$$\text{OAC}=\frac{V_{2}-V_{1}}{100\times 1.0}$$

(where V_1_ and V_2_ are the initial and final volumes, respectively)

### Foaming capacity and stability

The foaming capacity and stability of the protein isolate was done using a modified method of Lin et al. (1974). Two grams (2 g) of the sample was dissolved in 50 mL of distilled water. A food mixer at a speed set for fast beating was used to enhance adequate mixing of the mixtures. The initial and final volume after whipping in a 100 mL graduated cylinder was recorded. The percentage increase in volume due to whipping was calculated according to the method of Lawhon et al. (1972). For foaming stability, the foam volumes in the standing cylinder after whipping were recorded at 0.5, 1, 10, and 120 min (Sosulski 1962).

### Emulsion Capacity

Emulsion capacity of the samples was determined using the method described by Aluko and Yada (1995). The sample (1 g) was blended in 50 mL of distilled water for 30 seconds using a blender at a high speed. Vegetable oil (2.5 mL) was continuously added while blending until properly mixed, this was set‐aside for 30 min. The emulsion capacity was evaluated by the amount of oil emulsified by 1 g of the sample.

$$\text{Emulsion capacity}=\frac{\text{Emulsion height x 100}}{\text{water height x 1}}$$

### Wettability

Wettability of the samples were measured as the time in seconds required by 1 g of the flour sample to be completely wet on the surface of water under laboratory conditions. This was determined using the methods described by Aluko and Yada (1995). A clean glass beaker (600 mL capacity) was filled with 500 mL of distilled water. This was placed on a retort stand with a clean test tube clamped in an inverted position over the water in the beaker, with a distance of 10 cm between the beaker and the mouth of the test tube. The clean position on the test tube and water in the beaker were both marked with masking tape. Subsequently, 1 g of the sample was weighted into marked test tube and its mouth covered with a thumb. This was cautiously converted over the water and clamped with the retort stand at the marked spot without removing the thumb. With the stopwatch set to read, the thumb was removed and the sample allowed falling into the water surface as the stopwatch was put on simultaneously. The flour sample was observed and the stopwatch stopped as soon as the last samples got wet.

### Statistical analysis

All values are presented as the mean ± standard error of three replicate analyses. For multiple comparisons, the data were analyzed by one‐way ANOVA followed by Tukey's post hoc test (GraphPad Prism. 5.03, La Jolla, CA). The differences were considered significant at *P* < 0.05.

## Results

### Proximate composition of the fermented, defatted, and protein isolate of *P. biglobosa* seeds

The results of the proximate analysis of fermented, defatted, and protein isolate of *P*. *biglobosa* seeds are presented in Table 1. It is apparent that the protein isolate had the highest ash (6.0%) and protein (73.3%) contents as well as the lowest carbohydrate (8.8%), fat (5.7%), and moisture (5.1%) content when compared to the value of the fermented and defatted samples. The fermented sample shows the highest energy value (470.5 kJ) and fiber content (9.5%) when compared to the protein isolate (381.7 kJ, 1.1%) and the defatted (366.7 kJ, 8.6%) samples, respectively.

**Table 1:** Proximate composition of the fermented, defatted, and protein isolate of *Parkia biglobosa* seeds

Parameters	Fermented *P. biglobosa* (%)	Defatted *P. biglobosa* (%)	Protein isolate (%)
Moisture	7.3 ± 0.30^b^	10.2 ± 0.11^a^	5.1 ± 0.11^c^
Ash	4.3 ± 0.30^b^	2.9 ± 0.12^c^	6.0 ± 0.13^a^
Fiber	9.5 ± 0.32^a^	8.6 ± 0.14^b^	1.1 ± 0.15^c^
Protein	32.4 ± 0.21^c^	46.6 ± 0.15^b^	73.3 ± 0.17^a^
Carbohydrate	21.4 ± 0.12^b^	22.7 ± 0.21^a^	8.8 ± 0.13^b^
Fat	25.1 ± 0.11^a^	9.0 ± 0.32^b^	5.7 ± 0.21^c^
Energy (kJ)	470.5^a^	366.3^c^	381.7^b^

Data were expressed as mean ± SE. Means with different superscript along the row differs significantly (*P* < 0.05).

### Functional properties of fermented, defatted, and protein isolate of *P. biglobosa* seeds

Table 2 depicts the functional properties of fermented, defatted, and protein isolate of *P*. *biglobosa* seeds. The protein isolate showed the highest oil absorption capacity (4.2%), emulsion capacity (82%), foaming capacity (18%), and foaming stability (46.0%) as well as the lowest, bulk density (0.36%) and swelling index (1.77%) when compared to fermented (1.6%, 45.0%, 10.0%, 12.0%, 0.48%, 3.25%) and defatted (2.0%, 54.0%, 14%, 33.0%, 0.38%, 3.69%) samples, respectively. The defatted sample shows the highest water absorption capacity (8 %) and wettability (26%) compared to the protein isolate (3.0%, 14%) and fermented sample (5.6%, 6.5%), respectively.

**Table 2:** Functional properties of fermented, defatted, and protein isolate of *Parkia biglobosa* seeds

Parameters	Fermented *P. biglobosa*	Defatted *P. biglobosa*	Protein isolate
Water absorption capacity	3.0 ± 0.21^c^	5.6 ± 0.22^b^	8.0 ± 0.21^a^
Oil absorption capacity	1.6 ± 0.12^c^	2.0 ± 0.25^b^	4.2 ± 0.15^a^
Foaming capacity	10 ± 0.208^c^	14 ± 0.15^b^	18.0 ± 0.15^a^
Foaming stability	12.0 ± 0.153^c^	33.0 ± 0.15^b^	46.0 ± 0.25^a^
Emulsion capacity	45.0 ± 0.252^c^	54.0 ± 0.12^b^	82.5 ± 0.15^a^
Wettability	6.5 ± 0.21^c^	26.0 ± 0.23^a^	14 ± 0.2^b^
Swelling index	3.25 ± 0.15^a^	3.69 ± 0.15^a^	1.77 ± 0.153^b^
Bulk density	0.48 ± 0.01^a^	0.38 ± 0.1^b^	0.36 ± 0.01^b^

Data were expressed as mean ± SE. Means with different superscript along the row differs significantly (*P* < 0.05).

### Mineral composition of fermented, protein isolate, and defatted *P. biglobosa* seeds

The minerals composition in mg/100 g of the fermented, defatted, and protein isolate of *P. biglobosa* seeds are presented in Table 3. The mineral contents in the protein isolate were significantly higher in magnesium and zinc content compared to the fermented and defatted samples.

**Table 3:** Mineral composition of fermented, protein isolate and defatted *Parkia biglobosa* seed

Parameter	Fermented *P. biglobosa* (%)	Deffated *P. biglobosa* (%)	Protein isolate (%)
Calcium	1.97 ± 0.34^c^	9.15 ± 0.45^b^	10.70 ± 0.07^a^
Copper	0.02 ± 0.01^b^	0.04 ± 0.02^a^	0.07 ± 0.10^a^
Iron	0.22 ± 0.10^b^	0.24 ± 0.09^a^	0.28 ± 0.12^a^
Potassium	2.17 ± 0.02^b^	4.24 ± 0.34^a^	4.03 ± 0.11^a^
Magnesium	2.30 ± 0.11^c^	4.54 ± 0.66^b^	4.98 ± 0.34^a^
Manganese	0.05 ± 0.09^c^	0.27 ± 0.02^b^	0.33 ± 0.03^a^
Sodium	49.78 ± 0.01^a^	19.46 ± 0.02^b^	19.49 ± 0.34^c^
Zinc	0.05 ± 0.02^c^	0.15 ± 0.03^a^	0.18 ± 0.01^a^
Na/K	25.26 ± 0.03^a^	2.13 ± 0.48^b^	1.82 ± 0.22^c^
K/Na	0.04 ± 0.10^c^	0.47 ± 0.03^b^	0.55 ± 0.01^a^
K/(Ca+ Mg)	0.86 ± 0.02^a^	2.02 ± 0.05^a^	0.26 ± 0.05^c^

Data were expressed as mean ± SE. Means with different superscript along the row differs significantly (*P* < 0.05).

### Antinutritional composition (g/100 g) of the fermented, defatted, and protein isolates of *P. biglobosa* seeds

The results of the antinutritional composition of the fermented, defatted, and protein isolates of *P. biglobosa* seeds are presented on Table 4. The results showed the presence of phytate and oxalate in negligible amount.

**Table 4:** Antinutritional composition (g/100 g) of fermented, defatted, and protein isolates of *Parkia biglobosa* seeds

Parameter	Fermented *P. biglobosa*	Defatted *P. biglobosa*	Protein isolate
Phytate	0.174 ± 0.02^a^	0.36 ± 0.03^b^	0.14 ± 0.03^c^
Oxalate	0.189 ± 0.01^a^	0.31 ± 0.04^b^	0.02 ± 0.01^c^

Data were expressed as mean ± SE. Means with different superscript along the row differs significantly (*P* < 0.05).

## Discussion

The nutritional and protective potentials of *P. biglobosa* seeds was assessed in this study through the comparative evaluation of the proximate, functional, minerals, and antinutrient composition of the fermented, defatted, and protein isolate of the seeds as part of exploration into its antidiabetic mechanisms. Proximate composition is an important criterion to determine the nutritional values and quality of food (Qayyum et al. 2012). The findings of this study show that the protein isolate contains higher protein content than the fermented and defatted samples. This is in agreement with similar reports from other studies (Al‐Numair and Ahmed 2008; Olawuni et al. 2012). The high protein content of the protein isolate sample could be attributed to its production processes which increase the composition and availability of protein in the finished product (protein isolate) by inactivating antinutritional factors.

Dietary proteins plays an important role in natural synthesis and maintenance of body tissues, enzymes, and hormones as well as other substances required for healthy functioning (Hayat et al. 2014). Bioactive extracts isolated from medicinal plants are believed to delay, prevent, or manage metabolic diseases especially diabetes and its related complications using diverse mechanisms which could be established through their potential to increase insulin sensitivity, free radical scavenging abilities, hypolipidemic, hypoglycemic, hypocholesterolemic, antioxidative, anti‐inflammatory, and inhibition of *α*‐amylase and *α*‐glucosidase activities (Ogunyinka et al. 2015). The present work is one of the series of studies trying to elucidate the mechanism of action by which protein isolate from *P. biglobosa* could delay or attenuate oxidative stress, while concurrently improves various tissues and organ functions affected by free radical damage in different metabolic diseases.

In like manner, the protein isolate also contain higher ash content when compared to the fermented and defatted counterparts. Generally, high ash content is an indication that the protein isolate contains abundant mineral content (Iqbal et al. 2012). This agrees with the findings of Dwiani et al. (2014). The moisture value of protein isolate was lower when compared to the fermented and defatted sample. This finding is in agreement with the investigation reported by Olawuni et al. (2012). The low moisture content of the protein isolate is desirable because high moisture content encourages the growth of bacteria and mold, which could reduce stability and shelf storage capability.

In certain cardiovascular diseases such as cancer, atherosclerosis, and aging, excess consumption of fat has been implicated (Antia et al. 2006). The fermented and defatted samples had higher fat content compared to the protein isolate. The low fat content of the protein isolate is desirable, because it could be consumed instead of animal protein that contained high fat content (Adeyeye 2013). Aside from low fat content, the protein isolate also contain low fiber content, which could be attributed to the processing method in which other nutrients have been removed. Poor fiber content in protein isolates has previously been reported (Ojukwu et al. 2012). On the other hand, the protein isolate contains low carbohydrate content. This could also be attributed to the processing method in which the isoelectric precipitate removed soluble carbohydrate. This agrees with the report of Dwiani et al. (2014). The low carbohydrate and fat levels could be beneficial to diabetic individuals with increased serum lipid levels.

The functional properties are important in appropriateness of nutrients especially in growing children (Omueti et al. 2009). The functional properties have been reported to be improved through processing methods such as fermentation, sprouting, cooking, and soaking (Jirapa et al. 2001; Yagoub and Abdalla 2007). Findings from this study reveals that the protein isolate possess better water and oil absorption capacity than the fermented and defatted samples. Similar result was published for the protein isolate from *Cajanus cajan* Olawuni et al. (2012). The high water and oil absorption capacity suggests the protein isolate comprises of high polar amino acid and high hydrophobic residue on the protein molecules, respectively. Therefore, the protein isolate could be used for food formulation such as baked products, sausages, cheese, meat extender, and soups (Olaofe et al. 1998).

Foamability is essential in maintaining constituency, texture, and appearance of food that requires leavening and aeration properties (Onimawo and Asugo 2004). The protein isolate possess a high foaming capacity and stability. This could be attributed to its high protein and low fat content. High protein content increases viscosity that encourages the formation of cohesive protein layers. Fat is widely used as foaming inhibitor that destroys air–water surface, therefore, making water insoluble (Zayas 1997). Interestingly, the protein isolate have high emulsion capacity compared to the fermented and defatted sample; this could imply that the protein isolate contain both polar and nonpolar amino acids which increases the interaction between both oil and water molecules. Proteins can functions as an emulsifier since they have both hydrophobic and hydrophilic properties that can interact with oil and water in food systems (Mao and Hua 2012).

Bulk density explains the packaging capacity of food material. The low bulk density of the protein isolate implies that less quantity of it would be packaged in constant volume, therefore, ensuring economic package. Furthermore, low bulk density is of high nutritional value as it promotes easy digestion of food materials; therefore, the protein isolate would be easily digested (Osundahunsi and Aworh 2002). The protein isolate exhibited higher swelling index than the other samples. This is consistent with protein isolate from *Phaseolus lunatus*, which have been previously reported to have high swelling index (Ojukwu et al. 2012). Swelling index determines the amount of water that would be absorbed and the degree of swelling within a stipulated time. Swelling index is influenced by temperature, water availability, carbohydrate, and protein Yellavila et al. 2015. The high swelling index of the protein isolate could be as a result of high protein content. Equally, the high wettability of the defatted sample compared to that of the fermented and protein isolate could be attributed to the processing method. This implies that the defatted sample get wet before other samples.

Minerals are important constituents of human diet as they serve as cofactors for many physiological and metabolic processes. Deficiencies of certain micronutrients correlate to diabetic complications (Mooradian and Morley 1987). Analysis of the mineral content of *P. biglobosa* reveals that the protein isolate is a richer source of mineral elements when compared with other samples; this is based on the values obtained for copper, iron, manganese, magnesium, and zinc in this study. The appreciable copper content of the protein isolate implies that it is a good source of copper. Copper plays a fundamental role in cellular metabolism as it serves as cofactors for various important enzymes to function properly as well as in the formation of hemoglobin, myelin, and melanin, where it has been identified to play an essential role. Added to this, copper may also act as an antioxidant or a prooxidant. As an antioxidant, it prevents free radical damage by scavenging or neutralizing excess free radicals (Osredkar and Sustar 2011). For example, it is essential for the catalytic role of superoxide dismutase (SOD) in cellular defense against superoxide radicals (Khan and Awan 2014). However, abnormal copper metabolism has been implicated in the pathogenesis of several chronic diseases, such as diabetes and its associated complications. These pathogenic conditions appear to be common in copper deficiency as well as in copper overload; therefore, a tightly coordinated regulation is required (Zheng et al. 2008).

The low Na/K ratio of the protein isolate is an indication that it may reduce the incidence of hypertension as it would not induce high blood pressure, which is the major cause of cardiovascular diseases (Du et al. 2014), while the high calcium content in the protein isolate implied that it could prevent bone diseases such as rickets, osteoporosis, and osteomalacia. Notably, calcium also enhances the effective use of iron in the system (Adeyeye 2013). Iron is another essential mineral present in the protein isolate. Although, its overload leads to toxicity (Liu et al. 2009), iron is required for a number of biological functions, including proper functioning of the immune system, electron transfer reactions, gene regulation, cell growth and differentiation as well as binding and transport of oxygen (Siddiqui et al. 2014).

Manganese, though in trace amount, was present in the protein isolate. Manganese plays an important role as a cofactor in several antioxidant enzymes and in the metabolism of carbohydrates, proteins, and fats. Added to this, it plays an essential role in insulin synthesis and secretion. Alteration in its metabolism has been implicated in pathogenic conditions associated with diabetes development (Khan and Awan 2014). el‐Yazigi and co‐workers reported low levels of manganese in the white blood cells of diabetics compared to nondiabetic controls (el‐Yazigi et al. 1991).

Our result reveals the presence of magnesium in the protein isolate. Magnesium is required for the action of more than 300 enzymes in the body, where it participates in several significant physiological functions in the maintenance of good health and glucose homeostasis. Added to this, it has been identified to play a significant role in the release of insulin and the maintenance of pancreatic *β*‐cells. Its deficiency has been implicated in insulin resistance and carbohydrate intolerance as well as diabetic complications and dyslipidemia (Piero et al. 2012; Akhuemokhan et al. 2013; Khan and Awan 2014). Zinc was also appreciably detected in the protein isolate. Zinc is known to play a crucial role in antioxidant defense in type 2 diabetic patients where it enhances reduction and neutralization of free radicals and acts as a cofactor of SOD, by modulating glutathione metabolism and metallothionein expression. Its deficiency has been implicated in a number of metabolic abnormalities such as impaired glucose tolerance, decreased pancreatic insulin content as well as insulin degradation (Piero et al. 2012; Cruz et al. 2015).

In this study, our results revealed the presence of low antinutrient value in the protein isolate when compared to the other samples. The essence of antinutrient comparison within the three (3) different samples in this study was to identify the effect of each processing method on the antinutrient values. Although antinutrient was removed after defatting process with butanol, it is interesting to note that there were still traces of phytate and oxalate in the protein isolate. The values detected are at a safe level that poses no danger in diets. It is believed that fermentation reduces antinutritional factors; therefore, the processing method may account for the result obtained. This is in agreement with what was reported previously for African locust beans (Ijarotimi and Keshinro 2012). Antinutrients are natural compounds that interfere with the bioavailability of nutrients by interfering with their absorption in the gastrointestinal tract. Phytate and oxalate are examples of antinutrients that interferes with some mineral components such calcium, iron, zinc, and magnesium by forming insoluble complexes (Ijarotimi and Keshinro 2012).

## Conclusion

In conclusion, this study has shown the proximate, functional, mineral, and antinutrient composition of the protein isolate from *P. biglobosa* as balanced and a rich source of macro‐ and micronutrients. The most remarkable finding in this study is that the protein isolate possesses a high protein content associated with low fat, carbohydrate, and antinutrient contents. Therefore, the protein isolate from *P*. *bilogbosa* seeds can be considered a good source of protein supplement which could be developed as a nutraceutical toward prevention, treatment, and management of chronic metabolic diseases especially diabetes and its associated complications.

## Conflict of Interest

There is no conflict of interest.
